# 3D Printed Pharmaceutical Systems for Personalized Treatment in Metabolic Syndrome

**DOI:** 10.3390/pharmaceutics15041152

**Published:** 2023-04-05

**Authors:** Abdulsalam A. Alqahtani, Mohammed Muqtader Ahmed, Abdul Aleem Mohammed, Javed Ahmad

**Affiliations:** 1 Department of Pharmaceutics, College of Pharmacy, Najran University, Najran 11001, Saudi Arabia; aamari@nu.edu.sa; 2 Department of Pharmaceutics, College of Pharmacy, Prince Sattam Bin Abdulaziz University, Al-Kharj 11942, Saudi Arabia; mo.ahmed@psau.edu.sa

**Keywords:** metabolic syndrome, personalized medicine, 3D printing techniques, drug delivery approaches, pharmaceutical products

## Abstract

The current healthcare system is widely based on the concept of “one size fit for all”, which emphasizes treating a disease by prescribing the same drug to all patients with equivalent doses and dosing frequency. This medical treatment scenario has shown varied responses with either no or weak pharmacological effects and exaggerated adverse reactions preceded by more patient complications. The hitches to the concept of “one size fits all” have devoted the attention of many researchers to unlocking the concept of personalized medicine (PM). PM delivers customized therapy with the highest safety margin for an individual patient’s needs. PM has the potential to revolutionize the current healthcare system and pave the way to alter drug choices and doses according to a patient’s clinical responses, providing physicians with the best treatment outcomes. The 3D printing techniques is a solid-form fabrication method whereby successive layers of materials based on computer-aided designs were deposited to form 3D structures. The 3D printed formulation achieves PM goals by delivering the desired dose according to patient needs and drug release profile to achieve a patient’s personal therapeutic and nutritional needs. This pre-designed drug release profile attains optimum absorption and distribution, exhibiting maximum efficacy and safety profiles. This review aims to focus on the role of the 3D printing technique as a promising tool to design PM in metabolic syndrome (MS).

## 1. Introduction

Metabolic syndrome (MS) is a cluster of metabolic abnormalities characterized by hypertension, central obesity, insulin resistance, and dyslipidemia [1]. Patients with MS are closely linked to an elevated risk of developing diabetes and cardiovascular disease (CVD) [2,3]. The prevalence of MS in the adult population ranges from 20 to 25%, and in children up to 19%, whereas in patients with type II diabetes mellitus, it can reach up to 80% [4,5,6,7,8]. The low levels of high-density lipoprotein (HDL) followed by abdominal obesity is one of the observed components of MS. The dominance of obesity and type II diabetes often parallels one of the consequences of MS [9]. Patients with MS are at high risk of cardiovascular disease and neurological disorders. According to a World Health Organization (WHO) report, about 422 million people are suffering from diabetes worldwide [10]. The literature review conceals that Saudi Arabia has the highest prevalence of diabetes and a high rate of obesity, affecting more than one-third of the adult population [11]. The other components of MS were also reaching rising heights in Saudi Arabia, the predisposing kingdom as one of the top countries with the highest prevalence of MS [12]. A study showed a 39.8% prevalence of MS in Saudi Arabia according to Adult Treatment Panel III criteria and 31.6% according to International Diabetes Federation criteria [13]. The treatment of MS aims to treat individual components of MS, including antihypertensive, hypoglycemic, and anti-diabetic drugs. With the current conventional medication system, patients with MS were exposed to multidrug therapies with frequent dosing. This exposes the patient with MS to high financial burdens.

Moreover, the conventional drug formulations are also regimented with “fit to all” dosage strength. Therefore, clinicians have limited options to fit the dose as per the patient’s requirements [14]. This limitation in dose calibration may lead drug treatment to sub-therapeutic or potentially toxic levels [15]. This scenario has led the focus of researchers toward the concept of PM to tailor the dose as per the individual patient requirement. To identify the individual patient requirement, a study on pharmacogenomics has been taken into consideration to assess the genetic variability of an individual patient and their response to medication. This factor has been recognized as the key to ensuring drug safety via PM. PM is considered to be more precise, safer, not expose the patients to unnecessarily high doses, efficacious with improved patient compliance, and cost effective [16]. 

## 2. Personalized Medicine and Its Significance in MS 

PM refers to the customization of drug treatment for a particular diseased condition according to a specific patient’s needs, characteristics, and preferences [17]. The PM approach replaces the concept of one size fits all with the “right drug” in the “right dose” for the “right patient” at the “right time” [18]. PM guides clinicians on an appropriate drug dose for a specific patient for a particular diseased condition. The significance of PM is to establish a better healthcare system targeting customized therapies. The concept of PM is based on each individual’s genome response to a particular drug concerning diet and lifestyle activities, which plays a vital role in disease management. Therefore, PM is a diagnostic, preventive, and treatment approach based on inter-individual genetic differences and their response, together with therapeutic effects, adverse effects, and allergic reactions [19]. It has been reported that the prevalence of adverse effects due to untailored therapy is 75% to 85% [20]. In many instances, fixed-dose products were manifested due to their inflexibility in dosage strength after marketing. For instance, Atenolol, an antihypertensive drug, was initially marketed as a 100 mg tablet; later, they were introduced in 50 mg and 25 mg as they were found more suitable for elderly patients [21]. Moreover, multiple drug therapy has also led to poor patient compliance and increased risk of medication error by geriatric patients [22]. To overcome this, multiple drugs can be combined or formulated as a single pill for customized dosing [23]. A research group has formulated a multi-active polypill by using a 3D printing technique for the patient with MS serves as a PM approach for elderly patients on poly medicine [24]. Similarly, a 3D printed patch has been developed for a chemotherapeutic agent, which exhibited controlled release implanted directly into a pancreatic cancer patient for customized delivery [25]. The use of biological magnetic nanoparticles with suitable load surface area combined with hadron therapy and magnetic fluid hyperthermia also serves as a potential alternative for the treatment of pancreatic cancer [26,27]. The future combination of these therapies with 3D technologies opens promising future avenues in the design of more efficient treatments not only for cancer but also for other human diseases.

### 2.1. Role of Pharmacogenomics/Metabolic Profiling to Determine Personalized Medicine

Drugs are being tested and approved clinically still, but there is no way to predict how they respond to the individual patient. Still, many patients are hospitalized and die due to adverse drug reactions [28]. A drug may be safe for many patients but may cause adverse drug reactions in a few because of inter-individual gene variability. Therefore, pharmacogenomics may be used to identify an individual genome response to a drug and a safe customized treatment could be provided according to individual metabolic profiling [29]. The current trends focusing on the human genome and metabolic pathways to study an individual gene response to the medication have leaped over a decade. This will revolutionize the conventional treatment approaches towards PM [19]. The US FDA has recognized this paradigm shift in drug treatment and has given a path toward the PM approach by approving some of the personalized products [30]. PM relies considerably on pharmacogenomics, which focuses on drug response to a disease at the molecular level. Pharmacogenomics leads to better identification and implications of genetic predispositions to provide better preventive measures, early diagnostics, and safe drug treatment with fewer adverse effects [31]. The pharmacogenomics data help pharmaceutical companies to identify a specific group of people with variability in gene response to the drug and develop a specific product according to their needs. Including pharmacogenomics data of the population in clinical trials helps to identify the prototype gene showing variability in drug response with poor efficacy or adverse drug reaction. These data could be further used to develop a particular dose according to their gene responses for the specific group of population, showing variability. Consequently, this also reduces the time and cost consumed in clinical trials and drug development process. Subsequently, the pharmacogenomics and PM provide an improved drug development process, better medication selection, with a safe dose. Randomized controlled studies can provide evidence in support of the clinical application of pharmacogenetics [32]. In an instance, warfarin retrospective studies have shown that pharmacogenetic testing for CYP2C9 and VKORC1 in patients leads to more accurate warfarin dosing than either fixed dose or the use of dosing tables on warfarin labels [33]. Similarly, azathioprine and mercaptopurine exhibited variable clinical effect with Thiopurine S-methyltransferase (TPMT) enzyme gene variants responsible for increased hematopoietic activity. The TPMT enzyme gene variability causes a decrease in the enzymatic activity and thus causes problems in selection of a safe dose of a drug. Leukemic children develop severe bone marrow toxicity if treated with mercaptopurine having inherited TPMT deficiency [34]. Moreover, 5-fluorouracil-induced toxicity is due to Dihydropyrimidine dehydrogenase (PDP) enzyme genotype low levels [35]. Another classic example of a personalized drug is Primaquine, which is used for the treatment of malaria, and had caused acute hemolytic anemia in certain individuals with variability in the G6PD gene. This calls for genotyping of individuals to be treated with Primaquine to see G6PD gene variants in order to limit the use of this drug [36]. In addition to this, another cited example of personalized drug is imatinib, used in the treatment of chronic myelogenous leukemia (CML). Imatinib should only be given to individuals developing “Philadelphia chromosome” due to the fusion of two genomic regions resulting in increased tyrosine kinase enzyme activity in individuals with tumors which is inhibited by imatinib [37]. Although pharmacogenomics is the root to personalized medicine, other factors, such as epigenetic modifications, environmental exposure and behavior and tissue biomarkers, should also be integrated and assessed to determine personalized medicine [38,39]. Different technologies to record patient-specific profiling have emerged, such as proteomics, DNA sequencing and imaging and radiology protocols, that provide inter-individual variability in disease associated factors. The interest in the field of identifying factors, such as genetic variability in an individual patient in order to prevent personalized disease, has led to the further advancement into personalized disease surveillance and strategies to develop early detection protocols, such as mutation-specific therapies, pharmacogenetic-based diagnosis, use of pharmacogenomics databases, theranostics, proteomics and biomarker profiling based diagnosis for disease identification and personalized treatment [40,41]. Moreover, pharmacokinetic modelling plays a key role in the development of personalized medicine. Physiologically based pharmacokinetic (PBPK) modeling utilizes physiological and physicochemical data to simulate physiological responses to predict pharmacokinetic profiling, whereas population-based pharmacokinetic modeling (popPK) is used to simulate dosage regimen based on drug exposure in patients for a specific time course and exposes variability in drug response to study pharmacokinetics at population level [42,43]. 

### 2.2. Role of 3D Printing Technology in the Design of Personalized Medicine

3D printing is an additive manufacturing technique that uses digital design to formulate patient-specific drug delivery systems. With the use of the 3D printing techniques, complex structures and designs could be fabricated to achieve customization [44]. This flexibility offers many strategic approaches for the research and development of drug delivery devices and systems. The approval of Osteofab Patient-Specific Facial Devices and Patient-Specific implants by the United States Food and Drug Administration (US-FDA) in 2016 fabricated via 3D printing techniques by Oxford Performance Materials are the success lanes towards the fabrication of personalized devices [45,46]. The application of 3D printing in personalized drug delivery has gained interest in the pharmaceutical sector through FDA approval of the Spritam^®^ 3D printed tablet of an antiepileptic drug, which has met the criteria of rapid disintegrating formulation with high drug loading capacity to achieve customized dosing [47]. In addition, Triastek received an Investigational New Drug (IND) approval from US FDA for a 3D printed drug product (T19) fabricated by Melt Extrusion Deposition (MED), which is another breakthrough in the development of pharmaceutical products [48]. T19 is designed as a chronotherapeutic drug delivery system to treat patients with rheumatoid arthritis (RA). The patients will take the tablet at bedtime, and the peak drug concentration will be achieved in the early morning hours due to the delayed release of the drug from the developed system. Thus, targeting the circadian symptoms of pain, joint stiffness, and dysfunction, which are more acute in the morning hours in patients with RA. A second IND approval by US FDA for the product T20 is a breakthrough for Triastek’s digital design formulation development process. T20 provides once-daily dosing for the treatment of cardiovascular and clotting disorders [49]. Moreover, Triastek recently received another third IND approval for their product T21, by US FDA to initiate clinical studies. T21 is a colon-targeted drug product designed for the site-specific delivery of drugs for the treatment of ulcerative colitis [50]. These US FDA approvals give the roadway to drug manufacturing companies making the 3D printing technique the future of pharmaceutical product development. A list of US FDA-approved 3D printed devices and drug products for personalized delivery were shown in Table 1. 

There are different types of 3D printing techniques have been reported for fabricating drug delivery systems aimed at customized treatment for patients. The technical flexibility offered by 3D printing provides new opportunities to design on demand, patient-specific products. One of the finest examples of this is fabrication of a self-supported tacrolimus lipid-based suppository without the use of a mold by using a semi solid extrusion-based 3D printer. The suppositories were fabricated in different size and dose for personalized delivery of a narrow therapeutic index drug [51]. Moreover, advancement in 3D printing has led to the emergence of 4D printing, a novel concept in which smart materials and smart designs are used to fabricate smart objects that have the ability to exhibit pre-determined change, such as shape, functionality and properties, in response to an external stimuli, such as pH, temperature, electric or magnetic field. The use of hydrogels and shape memory polymers that respond to an external stimulus, such as solvent, temperature, pH and UV radiation, and results in change in configuration. Moreover, 4D printing, due to its peculiar feature, could be applied for drug targeting and bespoke treatment. An example of 4D printing is fabrication of bilayered hydrogel-based micro robots comprising of a pH responsive layer and magnetic responsive layer for anti-cancer therapy. The magnetic responsive layer facilitates transport of a fabricated system to the target site via external magnetic field, and the pH responsive layer delivers drug to the targeted site where the acidic nature of the tumor cells acts as a stimuli. Thus the 4D printed system provides decreased cell viability by 70% and avoids systemic circulation of the drug, thus reducing systemic side effects [52]. 

## 3. Types of 3D Printing Techniques Utilized in Product Development

The major types of 3D printing techniques used for pharmaceutical product design include inkjet 3D printing [53,54], including advanced electrohydrodynamic inkjet 3D printing technology [55], binder jet 3D printing [56,57,58], direct ink writing (DIW) [59,60,61], fused deposition modeling (FDM) [62,63,64], pressure-assisted micro syringes (PAM) [51,65], and laser-based 3D printing techniques, which includes stereolithographic (SLA) [66,67] and selective laser sintering (SLS) [68,69]. The various types of 3D printing techniques, parameters, and material choices are shown in Table 2.

**Table 2:** Different types of 3D printing techniques and materials utilized in product development.

3D Printing Technique	Category	Feed Material	Polymers/Excipients Used	Other Components	Ref.
FDM	Extrusion based	Drug loaded filament extruded through nozzle	Thermoplastic materials, such as PLA, PVA. Polymers, such as HPMC, Eudragit, and PVP.	Lubricant, filler and plasticizer. E.g., TEC, Talc, PEG 400, mannitol.	[70,71]
PAM	Extrusion based	Formulation paste filled in syringe extruded through nozzle	PVP, HPC, HPMC, EC, Carbopol, PVA, PEG.	Fillers, binders and disintegrants. E.g., mannitol, sodium starch glycolate, avicel.	[72,73]
Inkjet printing	Liquid based	Ink jetting on substrate	PLGA (poly lacto-co-glycolic acid) and PLA (poly-L-lactide), PEG/HPMC.	Substrate material: HPMC. PVA-CMC. Ink material: PEG 400, glycerol, ethanol.	[74]
Binder jet printing	Powder based	Binder jetting	PEO, Eudragit, PVP, EC, HPMC, mannitol, maltitol, maltodextrin	Binder: PCL, PVP K25, PVP K30.	[75]
SLA	Liquid based	Vat—photo polymerization	Monomers: polyethylene glycoldiacrylate (PEGDA), poly(propylene fumarate): diethyl fumarate (50:50), Dental SG resin, Elastic Resin	Photoinitiator: diphenyl(2,4,6-trimethylbenzoyl) phosphine oxide, Irgacure 184 (1-Hydroxycyclohexyl phenyl ketone)	[71,76]
SLS	Powder based	Laser-powder bed fusion.	PEO, Eudragit EPO, PVA, PEG, Kollicoat MAE, Kollicoat IR, Kollidon VA64, Eudragit RL, HPMC, Ethylcellulose,	Lactose monohydrate and microcrystalline cellulose, silicon dioxide, talc, Stearic acid, mannitol, cyclodextrin	[71,77]

### 3.1. Inkjet Printing

Inkjet printing involves the deposition of liquid droplets on a substrate in a continuous stream using either a thermal or a piezoelectrically driven nozzle. Inkjet printing is associated with some drawbacks, such as nozzle blockage and appearance of satellite droplets. These detrimental drawbacks can be overcome by flow modification, by use of inks with low impurities, by using smoothed nozzle internal surfaces and by optimizing waveform designs for enhancing the droplet quality [78,79]. Inkjet printing is also allied with photo-initiated UV curing to harden the printed objects [80]. A detailed description of inkjet-based 3D printing systems has been discussed in our previous review [44]. Inkjet printing is used to design various drug delivery systems, which include orodispersible films (ODF) of enalapril maleate and propranolol hydrochloride [81,82]. The inkjet printing technique is also applied for the coating of microneedles for transdermal drug delivery of insulin [83]. Apart from these, self-nano-emulsifying drug delivery systems were also developed by using drop-on-demand inkjet printing techniques [84]. Inkjet printing allied with UV photocuring was applied to design carvedilol tablets [80]. Aerogel microspheres and mesoporous silica nanoparticles were also developed using the inkjet printing technique [85,86]. The electrohydrodynamic (EHD) inkjet printing technology involves the high-voltages between the nozzle and the substrate creating an electric field, which allows the deposition of the ink [55]. Similarly, the direct ink writing (DIW) technique, which has been shown efficient to create functionalized lignin particles, could exhibit tunable antioxidant properties. This aspect is crucial to develop antimicrobial and antibacterial pharmacological treatments [59,60]. 

### 3.2. Binder Jet Printing

The first FDA-approved 3D printed product (Spritam^®^) was designed by using the binder jet printing technique. Binder jet printing is a drop-on powder deposition technique using either a thermal or a piezoelectrically driven nozzle. A binder liquid droplet was deposited on the powder bed to build a solid structure. Further, 3D printed tablets, devices, implants, and microparticles of various APIs with different sizes, shapes, and release profiles were designed by using binder jet printing techniques [87,88,89]. Binder jet printing is used to design personalized dosage tablets [90], fast disintegrating tablets [91], pulsatile release [92], delayed release [87], and linear release profile drug delivery systems [88]. One of the major advantages of binder jet printing is that it produces highly porous products that show rapid disintegration in a few seconds. In an instant, the drop-on powder deposition technique was used to design a captopril tablet for rapid dispersion of the drug using polyvinyl pyrrolidone (PVP) K25 as the binder ink. In this process, mannitol, maltitol, maltodextrin, and PVP were used as powder additives. The product with maltitol showed the shortest flash time of 6.09 ± 1.06 s [88]. 

### 3.3. Fused Deposition Modeling (FDM) 3D Printing

FDM is the most commonly employed 3D printing technique for pharmaceutical product design. In this technique, thermoplastic filaments were used as the feed material. They were extruded through an FDM-based 3D printer at the filament’s melting temperatures, resulting in layer-by-layer deposition of a 3D-designed object. The drug-loaded filaments were fabricated by using the hot melt extrusion (HME) technique. The polymer blends, APIs, and other additives were extruded through a screw-based extrusion system, which works on a motor-driven barrel heat to melt the blend and extrude it through a nozzle as filament. FDM is the most inexpensive, reproducible technique with high printing accuracy. Among all the 3D printing techniques, FDM is the most extensively used technique, and more than 80% of the 3D printed drug products are designed by using the FDM technique. A variety of polymers viz polyvinyl alcohol (PVA), polylactic acid (PLA), polyvinylpyrrolidone (PVP), hydroxypropyl cellulose (HPC), hydroxypropyl methylcellulose (HPMC), ethylcellulose (EC), hydroxypropyl methylcellulose acetate succinate (HPMCAS) and various grades of Eudragit has been employed to fabricate filament for FDM printing. Various dosage forms in the form of a tablet, capsules, floating devices, mucoadhesive films, suppository shells, vaginal rings, and acne masks were developed by using FDM 3D printing technique. Various drugs used in the management of MS were fabricated in customized shapes and sizes and characterized with tuned release profiles by using the FDM technique. The FDM-based 3D printed APIs used in the management of MS include amlodipine besylate [93], captopril [94], carvedilol [95], Felodipine [96], glimepiride [97], glipizide [98], lisinopril dehydrate [93], ramipril [99], Glibenclamide [100] and metformin hydrochloride [97].

### 3.4. Pressure-Assisted Microsyringes (PAM) 3D Printing

PAM 3D printing technique works based on the extrusion of semisolid material or paste through a syringe nozzle to build a layer-by-layer three-dimensional structure. The extrusion of paste was governed by pneumatic pressure or mechanical piston. A semisolid paste of APIs with suitable pharmaceutical-grade tableting excipients was fed into the extruder syringe to create desired 3D geometries [101]. Many researchers developed complex drug delivery systems with tunable release characteristics using PAM-based 3D printing techniques. The literature review revealed that a bi-layered tablet, polypill containing multiple drugs, floating device, self-nano-emulsifying drug delivery system (SNEDDS) tablet, and a capsular shell to alter the release characteristics of conventional tablet was designed by using the PAM 3D printing technique. Various APIs, including nifedipine, captopril, glipizide [102], pravastatin, atenolol, ramipril [24], propranolol [103], glimepiride [104], rosuvastatin [105] and dapagliflozin [106], used for the treatment of dyslipidemia, hypertension, and diabetes, were fabricated by using the PAM 3D printing technique. 

### 3.5. Stereolithography (SLA)

SLA is a laser-based 3D printing technique, which involves the solidification of photo-curable polymer resins by photopolymerization using a UV light source. The photocuring of liquid resin polymers was carried out layer by layer, resulting in the solidified 3D object. In the SLA technique, photopolymerization was performed by either cationic or radical polymerization. This technique has the advantage of non-thermal printing suitable for thermolabile drugs. The 3D-printed objects with elegant geometries display high resolution and require less printing time. However, the photo polymerizing resins should be selected with care due to the lack of biocompatibility and the possibility of API degradation. A multilayered poly print was fabricated containing four antihypertensive drugs (amlodipine, atenolol, hydrochlorothiazide, and irbesartan) by using the SLA technique, which resulted in an unexpected drug reaction between amlodipine and the polyethylene glycol diacrylate (PEGDA) photo polymer used [107]. SLA technique has been employed to develop modified-release tablets with different geometries [108,109], hydrogels [110,111], microneedles for transdermal delivery [112], implants [113], and anti-acne masks [114].

### 3.6. Selective Laser Sintering (SLS)

SLS is another laser-based 3D printing technique that involves laser sintering of powder particles, which builds layer-by-layer 3D objects via a thermal binding process. SLS is a single-step printing technique that forms high-resolution 3D objects with tuned geometries and modified release characteristics. The SLS technique has not been widely used due to high laser energy that may result in the API’s degradation. SLS has been employed to develop drug delivery devices [115], amorphous solid dispersions [116], orodispersible printlets [117], miniprintlets [118], and polyprintlets [119].

## 4. Drug Delivery Approaches for Treatment of MS Utilizing 3D Printing Techniques

Different types of 3D printing techniques were utilized to deliver single or multiple drugs for the management of MS-associated hypertension, hyperglycemia, and dyslipidemia as a customized medicine. 

### 4.1. PAM-Based 3D Printing Technique Utilized in Drug Delivery of MS

**Figure 1:**
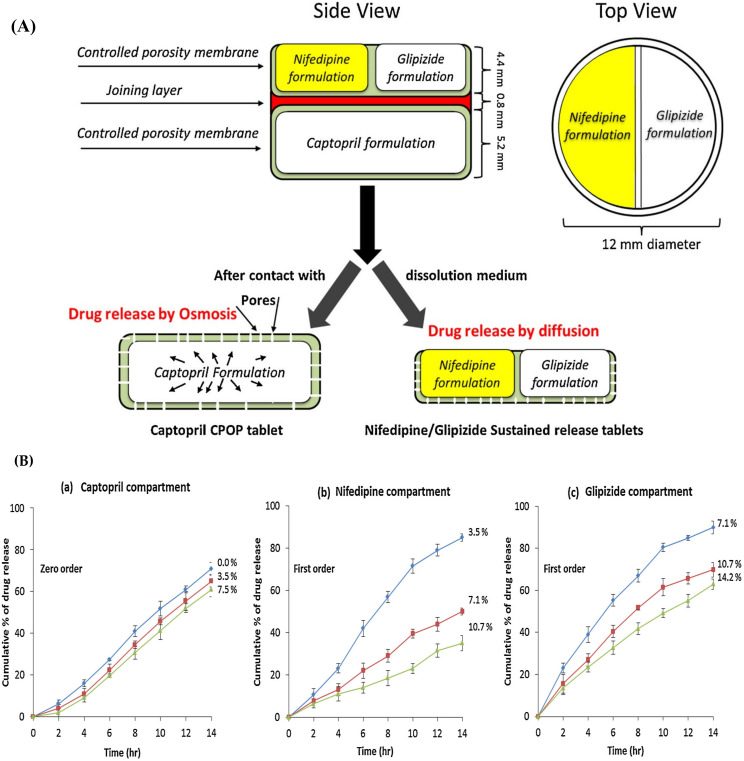
Schematic illustration of the concept of geometry for the design of multiple drug-loaded 3D printed tablets (**A**) and drug release profiles of captopril (representing 3 formulations in blue, red and green color containing 0.0%, 3.5% and 7.5% of HPMC 2208, respectively), nifedipine (representing 3 formulations in blue, red and green color containing 3.5%, 7.1% and 10.7% HPMC 2208, respectively), and glipizide (representing 3 formulations in blue, red and green color containing 7.1%, 10.7% and 14.2% HPMC 2208, respectively) from different compartments follow different release kinetics (**B**). Reprinted with permission from Ref. [102]. Copyright 2015 Elsevier.

A research group has demonstrated an extrusion-based 3D printing technique to develop multiple drug-loaded 3D printed tablets with modified release profiles. The multipill consists of an osmotic release compartment containing captopril and a sustained release compartment with drugs nifedipine and glipizide. HPMC 2208 is used as hydrophilic matrix in varying concentration to control the drug release from each compartment. The captopril-HPMC formulation feedstock contains lactose and sodium chloride which acts as an osmogen and microcrystalline cellulose (MCC), which acts as an squeezing and pushing agent. Similarly, the nifedipine and glipizide–HPMC formulation feedstock composed of PEG 6000, tromethamine which acts as solubilizer, and lactose which acts as a filler. The joining layer of the two compartments was composed of croscarmellose sodium (CCS) and sodium starch glycolate (SSG), which acts as a disintegrant, polyvinylpyrrolidone (PVP K30), which acts as a binder, and D-mannitol, which acts as a filler in the feedstock. The concept was to deliver a customized drug dose and drug release for a hypertensive patient with diabetes. The 3D printing was carried out by using an extrusion-based 3D printer with pharmaceutical-grade common tableting excipients. The drug release studies revealed the sustained release of the compartments for 14 h. Zero-order release kinetics were attributed to the captopril compartment and first order and Korsmeyer–Peppas release kinetics by nifedipine and glipizide portions, respectively [102]. The geometry of this multiple drug-loaded 3D printed tablet and drug release profile was represented in Figure 1.

**Figure 2:**
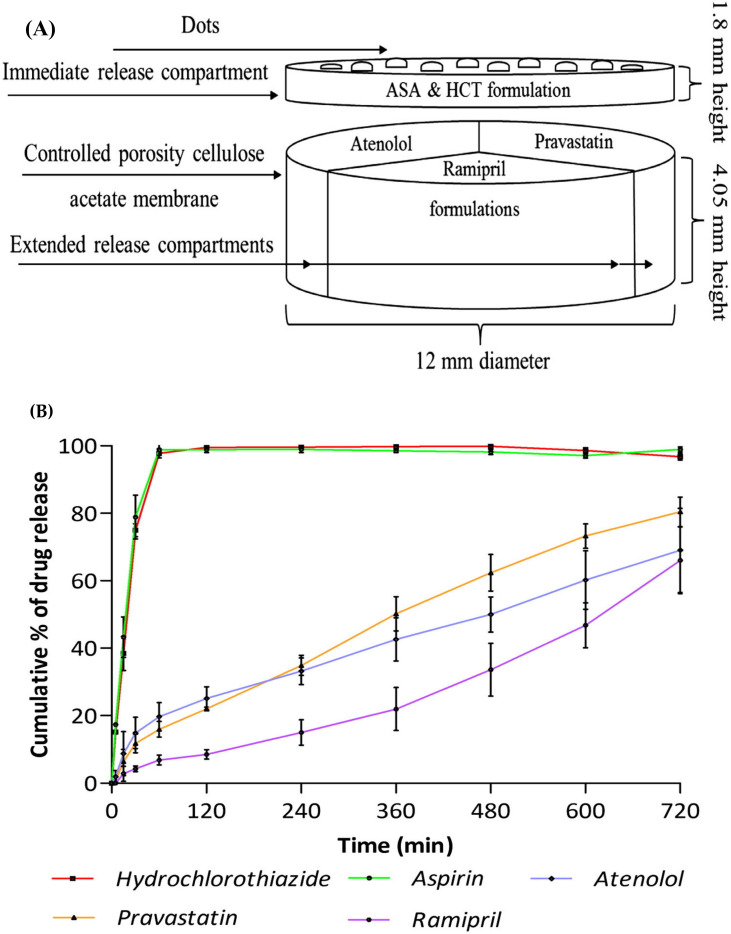
Schematic illustration of the concept of geometry for the design of 3D printed polypill (**A**) and drug release profile of hydrochlorothiazide, aspirin, atenolol, pravastatin, and ramipril from polypill (**B**). Reprinted with permission from Ref. [24]. Copyright 2015 Elsevier.

The same research group developed a polypill comprising five compartments, of which, two immediate releases and three sustained releases. The immediate release compartment contained aspirin and hydrochlorothiazide, and sustained release compartments were filled with pravastatin, atenolol, and ramipril. HPMC 2208 is used as a hydrophilic matrix for the sustained release compartment, whereas sodium starch glycolate and polyvinylpyrrolidone (PVP K30) were used in the immediate release compartment as a disintegrant and binder, respectively. This investigation aims to provide a single pill for patient compliance and to customize the dose and release profiles of the drug regimen. With the 3D printing technique, complex drug regimens can be combined in a single pill, achieving modified release profiles according to individual needs. The 3D printing was carried out by using a PAM-based extrusion 3D printer (regenHU 3D printer). The drug release studies showed aspirin and hydrochlorothiazide drug compartment achieved an immediate release of 75% within 30 min, whereas a sustained release of 69%, 81%, and 66% for 720 min was achieved by pravastatin, atenolol, and ramipril compartment, respectively [24]. The geometrical arrangement of this polypill and its release profile is represented in Figure 2.

Similarly, our research group has fabricated a self-nano-emulsifying 3D printed tablet of an anti-diabetic drug (dapagliflozin). This investigation aimed to fabricate a solid 3D printed SNEDDS tablet in three sizes with three different doses for personalized dosing. The 3D printed SNEDDS tablet was developed by using an extrusion-based 3D printer (Biobot 1). The liquid SNEDDS system was developed as a 3D printable paste by incorporating poloxamer 188 which acts as an emulsifier and solidifying agent in the SNEDDS system along with PEG 6000 which act as a solidifying agent. The developed nanosystem exhibited enhanced solubility of dapagliflozin in the self-nano-emulsifying system. The 3D-printed SNEDDS tablet showed immediate drug release of more than 75% within 20 min. Thus, the developed SNEDDS system, coupled with 3D printing, attributes an improved biopharmaceutical profile for the poorly water-soluble drug with personalized dosing [106].

Another research group developed a SNEDDS-based 3D printed tablet to enhance the aqueous solubility and dissolution characteristics of glimepiride. In this study, three different manufacturing techniques were used to develop glimepiride tablets via SNEDDS-based 3D printed tablet, SNEDDS-based liquisolid tablet, and directly compressible tablet. The 3D printed tablet was fabricated by using an extrusion-based 3D printer. Black seed oil-based SNEDDS were developed. The developed SNEDDS showed a droplet size of 45.607 ± 4.404 nm and attributed the glimepiride solubility of 25.002 ± 0.273 mg/mL. The 3D printed glimepiride tablet was fabricated by using HPMC as polymeric matrix to form a paste. A mixture of Neusilin, fujiSil and avicel is added to the formulation as an adsorbent. PVP K90 and Ac-Di-Sol were incorporated as a binder and disintegrant, respectively. The 3D printed glimepiride tablet revealed an extended drug release profile of 12 h. The drug release shown by the liquisolid tablet was faster within 120 min in comparison to the directly compressible tablet. The in vivo pharmacokinetic study revealed relative bioavailability of 121.68% and 113.86% by 3D printed tablet and liquisolid tablet, respectively. Thus, the aforementioned investigation provides a promising technique to enhance the solubility, dissolution profile, and in vivo pharmacokinetic behavior of a poorly water-soluble drug [104].

In another instance, a SNEDDS-based multicompartment 3D printed tablet was fabricated containing glimepiride and rosuvastatin for customized dosing to the hyperlipidemic patient with diabetes. In this investigation, Curcuma oil-based SNEDDS with between 80 and PEG 400 were developed. HPMC polymeric gel was prepared for 3D printing of liquid SNEDDS. To the prepared gel tableting excipients, such as avicel, methocel and lactose, were added, which acts as a adsorbents, PVP K90 and Ac-Di-Sol were incorporated, which act as a binder and a disintegrant in the formulation paste for 3D printing. The 3D printing was carried out by using an extrusion-based 3D printer (REGEMAT3D V1 BioPrinter, REGEMAT Inc. Granada, Spain). SNEDDS-based and non-SNEDDS-based 3D printed tablets were fabricated where the dose of glimepiride and rosuvastatin can be modified according to personalized use. The drug release studies showed superior release characteristics from SNEDDS-based 3D printed tablets in comparison to non- SNEDDS-based 3D printed tablets. The in vivo pharmacokinetic study showed a relative bioavailability of 159.50% and 245.16% for glimepiride and rosuvastatin, respectively. Thus, this study provides a proof of concept to develop multiple drugs containing 3D-printed tablets for customized delivery to treat MS [105]. The various drug delivery approaches in the management of MS using PAM-based 3D printing techniques are summarized in Table 3.

3D printing techniques have also been utilized to fabricate drug delivery devices, which enables tailored drug release from tablets fabricated by conventional methods. In an instance, a 3D printed coating shell was designed by our research group to encapsulate an immediate release conventional marketed tablet of propranolol hydrochloride to tailor the drug release from the enclosed tablet. An extrusion-based 3D printing technique was employed to fabricate the control release shell. The formulation paste to develop a control release shell via 3D printing process was composed of cellulose acetate, d-mannitol and PEG 6000, which acts as a rate controlling polymer, pore forming agent and plasticizer, respectively. The enclosed immediate release tablet exhibited a modified drug release profile, thus providing a solution to tune the drug release characteristics of the tablets fabricated by conventional methods [103].

### 4.2. FDM-Based 3D Printing Technique Utilized in Drug Delivery of MS

**Figure 3:**
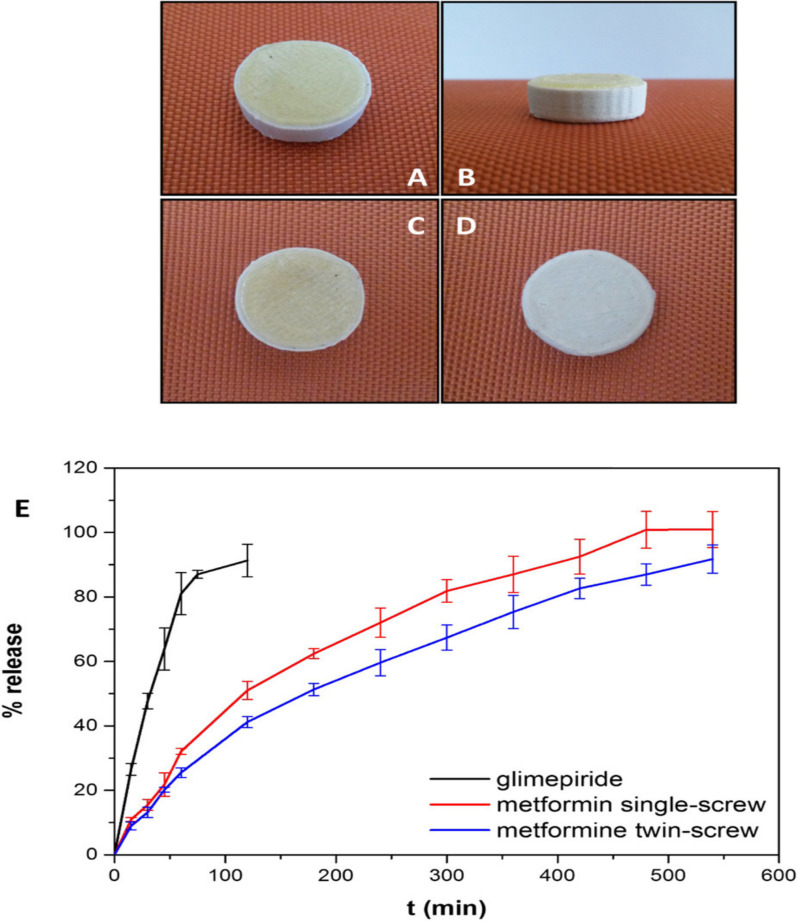
Schematic illustration of 3D printed bilayered tablet. (**A**) Front view. (**B**) Side view. (**C**) Top view. (**D**) Bottom view. (**E**) Drug release profile of glimepiride, metformin (single screw), and metformin (twin screw) from 3D printed bilayer tablet. Reprinted with permission from Ref. [97]. Copyright 2018 Elsevier.

A research group for customized delivery of metformin and glimepiride in diabetic patients fabricated an FDM-based bilayered 3D printed tablet. In this work, metformin was formed as a sustained release layer, and glimepiride was incorporated as an immediate release layer. Eudragit RO and PVA were employed to develop drug-loaded sustained release and immediate release filaments, respectively. The filament was developed by using a Filabot Original^®^ single-screw extruder (Filabot Inc., Montpelier, VT, USA) and the optimized filament formulation was extruded through a co-rotating twin-screw HAAKE MiniLab^®^ extruder (Thermo Scientific, Waltham, MA, USA). The 3D printing was carried out by using an FDM-based 3D printer (MakerBot Replicator^®^ 2× 3D printer, MakerBot Inc., New York, NY, USA). The filaments were characterized for extrudability, and printability. A printing accuracy ranging between −100, and +200 μm was revealed by Microfocus computed tomography (μCT) imaging. The incorporated drug showed amorphous dispersion revealed by X-ray (XRD) diffractograms. The results of the drug release studies showed an immediate release in 75 min for the glimepiride and a sustained release in 480 min for metformin. Thus, this study proves the capability of 3D printing to incorporate two APIs in situ for personalized delivery according to an individual patient’s needs. The concept of multiple drug incorporation in a single pill improves patient compliance by reducing the frequent dosing and cost of treatment [97]. The geometry of the bilayered 3D-printed tablet and drug release profile is shown in Figure 3.

**Figure 4:**
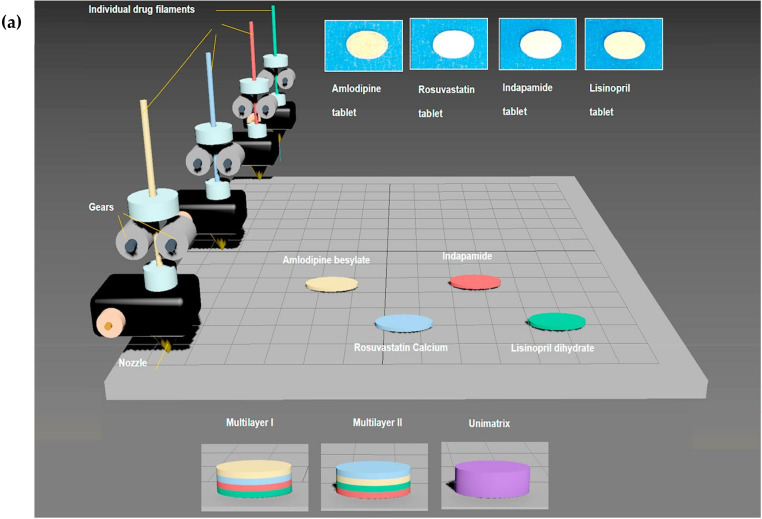

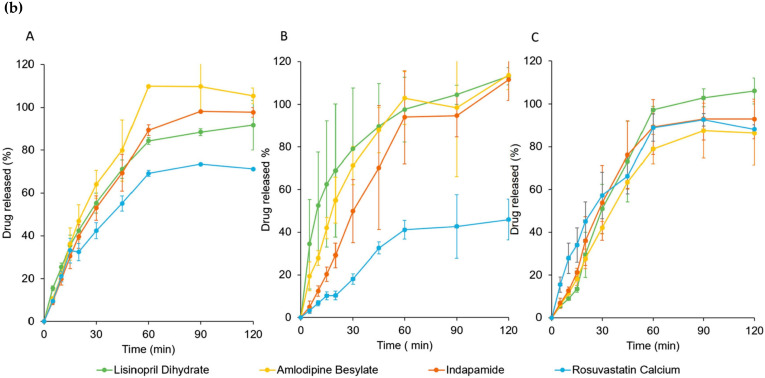
Schematic illustration of a concept of the geometrical arrangement of a multilayered 3D printed tablet (**a**) and drug release profiles of each drug from multilayer I tablet (A), multilayer II tablet (B), and unimatrix tablet (C) (**b**). Reprinted with permission from Ref. [93]. Copyright 2019 Elsevier.

In an approach, a research group fabricated a multilayered polypill unit to facilitate fixed-dose combinations for a complex multidrug treatment regimen. In this approach, an FDM-based 3D printing technique was used to develop polypill with a PVA-based unimatrix and multilayered architecture. Four model drugs were incorporated, i.e., lisinopril dihydrate, indapamide, amlodipine besylate, and rosuvastatin calcium for customized dosing for the treatment of cardiovascular disease. The PVA-based drug-loaded filaments were extruded using a Thermo Scientific HAAKE MiniCTW hot melt extruder. In the filament extrusion process, distilled water is added as a temporary plasticizer that resulted in lowering the extrusion temperatures and 3D printing temperatures from 170 °C and 210 °C, to 90 °C and 150 °C, respectively. This novel approach of using distilled water in the filament-making procedure reduced the thermal stress on the components and resulted in low-temperature extrusion and printing processes. The concept of using different matrices for each drug prevents incompatibility and provides flexibility in dose customization. Makerbot 2x 3D printer was used for the printing of the unimatrix and multilayered oval-shaped design tablets. The drug release from the multilayered tablet for each drug depends on the position of the drug layer in the tablet. The multilayered tablet exhibited biphasic drug release showing faster drug release for the outer layers and initial slow drug release for the inner layers, which became rapid after a lag time of 15 min. Thus, by fabricating a multilayered polypill, a customized dose and drug release can be achieved by altering the drug layer positions in the polypill [93]. The geometrical arrangement of multilayered 3D printed tablets and the drug release profiles were represented in Figure 4.

Moreover, another research group demonstrated the fabrication of a duo tablet for the controlled delivery of glipizide using FDM-based 3D printing technique. The duo tablet was made by using a drug-loaded PVA filament. The filament extrusion was carried out by using an LSJ20 single-screw extruder. The design of the duo tablet is composed of a tablet embedded within a large tablet loaded with different drug concentrations. The 3D printing was carried out by using a dual nozzle 3D printer, ClouovoDelta-MK2. The results of the drug release studies revealed a 5-h controlled and delayed release behavior of glipizide due to different drug concentrations in each tablet. A 90% drug release was achieved in the first two hours from the external tablet, and the drug release from the internal tablet was commenced after a lag time of 85 min. This delayed release behavior by the internal tablet is dependent on the external tablet thickness and polymer composition. The drug release kinetics exhibited a Korsmeyer–Peppas pattern. Thus, a controlled release 3D printed device was fabricated by altering the concentration of the drug via hot melt extrusion [98].

Another research group demonstrated the fabrication of a channeled 3D printed tablet of metformin hydrochloric acid using an FDM-based 3D printing technique. This approach loaded PVA filament with the drug by a modified solvent diffusion method. The drug-loaded filament was printed as a channeled tablet with 10% infill using a Makerbot experimental 2× dual extruder printer. The channeled design increased the surface area for drug dissolution. The dissolution studies showed more than 95% of drug release was attributed after 180 min. In this approach, the drug loading was performed by filament soaking in contrast to the high-cost hot melt extrusion process. Thus, the 3D printing technique provides a roadway towards various tablet designs, customized dosing, and modifying the release characteristics of the APIs [120].

In another instance, a research group demonstrated the applicability of 3D printing to fabricate fixed-dose combinations of drugs to a dynamic dose combiner according to individual patients’ needs. In this research, a bi-layered tablet of two antihypertensive drugs was designed in different combinations. Enalapril maleate and hydrochlorothiazide were loaded in different concentrations using Eudragit EPO filament. The extrusion was carried out by using a HAAKE MiniCTW hot melt extruder. The thermal analysis and X-ray diffractometric studies of the extruded filament represented the presence of amorphous enalapril maleate and crystalline hydrochlorothiazide in a polymeric matrix. The bi-layered tablet was printed by using an FDM-based 3D printer Makerbot Replicator 2X. The bi-layered tablet was designed with six different dose combinations. The dissolution studies indicated a similar drug release pattern for three sets of the bi-layered tablet. The release was governed by erosion of the polymeric matrix irrespective of the physical form of the two-layered drugs. Thus, this approach provides a shred of evidence that dynamic dose dispensing can be achieved for fixed-dose combinations according to the patient’s need [121].

### 4.3. Inkjet and Laser-Based 3D Printing Technique Utilized in Drug Delivery of MS

A research group fabricated a solid dispersion tablet for a poorly water-soluble drugs via inkjet 3D printing. In this study, a poorly water-soluble drug, carvedilol, was printed into different geometries (cylinder, ring, mesh, and thin film) using a photocurable N-vinyl-2-pyrrolidone (NVP) and poly (ethylene glycol) diacrylate matrix by UV curable inkjet 3D printing technique. The 3D-printed tablet showed more than 80% of drug release within 10 h. The release profile was indicative of the surface area to volume ratio. Among the different geometries, the thin film showed the fastest drug release, followed by the ring and mesh design, and the slowest drug release was exhibited by the cylinder-shaped tablets. Thus, this approach provides evidence that by altering the geometry of the 3D-printed tablet, a variety of drug release profiles can be gauged [122].

Another research group demonstrated a polypill for combination therapy with photocurable bioinks by using the inkjet 3D printing technique. In this study, lisinopril and spironolactone were loaded into hyaluronic acid and poly (ethylene glycol) (PEG) photocurable bioinks and printed into a preform multicompartment tablet fabricated via binder jetting 3D printing technique. The printed bioinks were photopolymerized with a customized dose. The 3D-printed tablet exhibited a sustained drug release profile. This approach combines binder jetting (powder bed 3D printing) with photocurable biomaterial jetting (inkjet 3D printing) to fabricate a 3D printed tablet with both hydrophilic and hydrophobic API’s [123]. 

A research group fabricated a 3D polyprintlet by using the SLS technique and developed a non-destructive dose quantification method for multiple drugs in a single 3D printed polyprintlet. In this study, polyprintlet film and cylinders were designed and loaded with different concentrations of two distinct drugs, amlodipine and lisinopril. The multiple drug-loaded polyprintlets were evaluated for dose content by using a portable near-infrared (NIR) spectrometer using the partial least squares (PLS) regression method. The developed validated calibration models showed excellent linearity, accuracy, and specificity for quantifying drugs. This approach provides a novel non-destructive dose quantification method for multiple drugs in a 3D-printed polypill [119]. 

Another research group fabricated transdermal polymeric microneedle insulin patches using the SLA 3D printing technique. In this study, cones and pyramid-shaped microneedles were fabricated using photopolymerized resin. Inkjet printing of insulin in Trehalose, mannitol, and xylitol as formulation carriers was carried out on microneedles. The selected carriers preserve the integrity and stability of insulin and provide a rapid release of insulin. The drug release from porcine skin using Franz cell was rapid within 30 min from both the microneedle designs. Thus, the 3D printing technique provides an effective way for transdermal delivery of insulin via microneedle patch [83]. The various drug delivery approaches exploited in the management of MS using different 3D printing techniques are summarized in Table 4.

## 5. Conclusions and Future Prospectives

The concept of 3D printing of pharmaceutical products at the point of care based on patient-specific personalized prescriptions revolutionizes the pharmacy compounding practice. Clinical university hospital Santiago de compostela, Spain, demonstrated the application of 3D printing technique for the first time to design personalized medicine. A chewable printlet of isoleucine was fabricated for the treatment of a rare metabolic syndrome, maple syrup urine disease (MSUD), in the pediatric population to improve patient acceptability and safety. The 3D printlets were fabricated in different sizes, doses and shapes and were intended to be chewed and swallowed without water. The chewable colored printlets in different sizes and flavors were well accepted by children and thus showed improved patient acceptability and compliance. The plasma drug concentrations exhibited optimum drug concentrations, thus making the 3D printing technique a reliable and accurate method for personalized dosing of a drug. The success story of this study provides a way to establish pharmacy-based drug compounding. However, the conventional pharmaceutical compounding does not follow good manufacturing practice (GMP) regulations, and is also associated with a few drawbacks, such as product quality and variability in dose strength. However, these limitations could be overcome by automation of compounding using 3D printers in a clinical pharmacy set up which works in a closed system, reduces contamination and produces personalized medicines with improved quality and dose accuracy. The potential of 3D printing technique is its ability to print any size, shape, color and flavor printlet according to a patient’s preference, thus improving patient acceptability and compliance. With 3D printers it is also possible to use specific excipients to avoid hypersensitivity reactions in specific patients. However, the potential issues in implementation of 3D printing in clinical practice is the lack of regulatory guidelines from FDA and other regulatory bodies to fabricate 3D printed personalized products in hospital pharmacies, lack of approved 3D printers and approved polymers that fabricate high quality products, cost affordability for personnel training, a range of printers set up in clinical settings, and lack of GMP guidelines to avoid cross contamination and batch to batch variability of printed products. Moreover, this concept of pharmacy-based dosage design is not practical and feasible to apply for all conventional therapies. A few categories of drugs, such as drugs with low stability, drugs that have low market value due to toxicity concerns, and drugs that show high inter-subject variability in therapeutic response and are anticipated for therapeutic drug monitoring (TDM), should be targeted to design patient-specific products. The concept of PM is evolving slowly and is restrained by legal provisions concerning privacy related to patient-specific pharmacogenomics data, regulatory requirements, cost, and practice implementation. However, 3D printing has paved the way to fill the gaps within the conventional treatment system and establish a secure and effective PM concept. The future perspective of PM is to provide a better healthcare management system, which seems to be possible with this technological advancement of 3D printing in pharmaceuticals.

## Figures and Tables

**Table 1:** List of various US FDA-approved 3D printed pharmaceutical products/devices.

Approval Status	Company	Product Name	Drug Delivery System/Device	Technique	Ref.
US FDA approved in market	Aprecia 3DP Pharmaceuticals Company	Spritam	Zip dose fast disintegrating tablet containing Levetiracetam, antiepileptic drug	Powder-based Binder Jet 3D Printing Technique	[47]
US FDA approved	Oxford Performance Materials Inc.	Osteofab	Patient-specific facial device	SLS	[46]
US FDA approved	Oxford Performance Materials Inc.	Osteofab	Patient-specific implants	SLS	[45]
Investigational New Drug (IND) approval by US FDA	Triastek Inc.	T19	Chrono therapeutic drug delivery system to treat patients with rheumatoid arthritis	MED	[48]
IND approval by US FDA	Triastek Inc.	T20	Once-a-day treatment for cardiovascular and clotting disorder	MED	[49]
IND approval by US FDA	Triastek Inc.	T21	Site-specific delivery of drugs for the treatment of ulcerative colitis	MED	[50]

**Table 3:** Various drug delivery approaches in the management of MS using PAM-based 3D printing technique.

Drug	Disease	Dosage Forms and Its Critical Attributes	Outcome	Ref.
Captopril, Nifidipine, glipizide.	Diabetic and hypertensive patient	Three drugs containing tablet (multipill) with defined drug release profiles for a complex dosage regimen	The designed multipill exhibited an osmotic and diffusion control sustained release of the drug for a period of 14 h.	[102]
Aspirin, hydrochloro thiazide, pravastatin, atenolol, and ramipril	Cardiovascular disease and high blood pressure	Five drugs in a tablet (polypill) with tailored drug combinations and release characteristic for complex dosage regimen	The designed polypill showed an immediate drug release of 75% within 30 min by aspirin and hydrochlorothiazide drug compartment, whereas a sustained release of 69%, 81%, and 66% for a period of 720 min was achieved by pravastatin, atenolol, and ramipril compartment, respectively.	[24]
Dafagliflozin	Diabetes	SNEDDS tablet with customized dosing and tuned drug release profile for poorly soluble drug.	The SNEDDS tablet exhibited an enhanced solubility and drug release of dapagliflozin and attributed to customized dosing.	[106]
Glimepiride and rosuvastatin	Hyperlipidemic patients with diabetes	Multicompartment 3D printed SNEDDS tablet for combined drug therapy	The multicompartment 3D printed SNEDDS tablets showed superior drug release characteristics in compare to liquisolid and directly compressible tablets. The relative bioavailability was found to be 159.50% and 245.16% for glimepiride and rosuvastatin, respectively.	[105]

**Table 4:** Different drug delivery approaches exploited in the management of MS using various 3D printing techniques.

Drugs	Disease	Dosage Form and Its Critical Attributes	3D Technique	Outcome	Ref.
Metformin and glimiperide	Diabetes	Tailored made bilayered tablet for combined pharmacotherapy	FDM	The 3D printed bilayered tablet showed an immediate release of glimepiride and a sustained release of the metformin.	[97]
Lisinopril, indapamide, amlodipine and rosuvastatin	Cardiovascular disease	Multilayered polypill for patient-centred therapy with orchestrating release profiles	FDM	The drug release from the multilayered tablet for each drug depends on the position of the drug layer in the tablet. The multilayered tablet exhibited biphasic drug release showing faster drug release for the outer layers and initial slow drug release for the inner layers, which become rapid after a lag time of 15 min.	[93]
Glipizide	Diabetes	Specialized designed duo tablet with variable drug concentration distribution for tuned drug release	FDM	The duo tablet exhibited controlled and delayed release behavior of glipizide due to the customized dose in each tablet. From the external tablet, 90% drug release was achieved in the first two hours and from the internal tablet, the drug release commenced after a lag time of 85 min.	[98]
Enalapril maleate and hydrochlorothiazide	Hypertension	Bi-layered tablet in a customized flexible dose combination	FDM	The bi-layered tablet designed with six different dose combinations indicated a similar drug release pattern for three sets of a bi-layered tablets. Thus this approach provides evidence that dynamic dose dispensing can be achieved for fixed-dose combinations according to the patient’s needs.	[121]
Amlodipine and lisinopril	Hypertension	Multidrug-loaded poly printlets with customized dosing characterisation	SLS	Provides a novel non-destructive dose quantification method for multiple drugs in a 3D-printed polypill.	[119]
Insulin	Diabetes	Microneedle patches for personalized transdermal delivery of insulin	SLA	The drug release from the designed microneedles was rapid within 30 min. The selected carrier preserves the integrity, stability of insulin, as well as provides a rapid release of insulin from cone, and pyramid shaped microneedles.	[83]

## Data Availability

Not applicable.

## Abbreviations

PM	Personalized medicine
MS	Metabolic syndrome
3D	Three-dimensional
HDL	High-density lipoprotein
CVD	Cardiovascular disease
IND	Investigational New Drug
MED	Melt Extrusion Deposition
US-FDA	United States Food and Drug Administration
FDM	Fused deposition modelling
PAM	Pressure assisted micro syringes
SLA	Stereolithographic
SLS	Selective laser sintering
ODF	Orodispersible films
PVA	Polyvinyl alcohol
PLA	Polylactic acid
PVP	Polyvinylpyrrolidone
HPC	Hydroxypropyl cellulose
HPMC	Hydroxypropyl methylcellulose
EC	Ethylcellulose
HPMCAS	Hydroxypropyl methylcellulose acetate succinate
SNEDDS	Self-nano-emulsifying drug delivery system
PEGDA	Polyethylene glycoldiacrylate
NVP	N-vinyl-2-pyrrolidone
PEG	Polyethylene glycol
μCT	Microfocus computed tomography
XRD	X-ray diffractograms
PEO	Polyethylene oxide
APIs	Active pharmaceutical ingredients.
